# Impact of Sars-CoV-2 pandemic on the Veneto Region multitissue bank activity

**DOI:** 10.1007/s10561-022-09997-1

**Published:** 2022-03-02

**Authors:** Giulia Montagner, Rudy De Vettor, Francesca Favaretto, Daniela Vici, Claudia Del Vecchio, Elisa Franchin, Diletta Trojan, Giuseppe Feltrin

**Affiliations:** 1 Fondazione Banca dei Tessuti di Treviso Onlus, Treviso, Italy; 2 Department of Molecular Medicine, University of Padova, Padua, Italy; 3 Regional Centre for Transplant Coordination, Padua, Italy

**Keywords:** Tissue bank, Covid-19, Pandemic, Amniotic membrane, Cardiovascular tissue, Musculoskeletal tissue

## Abstract

Covid pandemic affected donation activities worldwide, especially for living donation due to the lack of elective surgery. Moreover, the number of heart-beating and non-heart beating donors has recorded a decrease. Fondazione Banca dei Tessuti di Treviso (FBTV) is a non-profit healthcare organisation, located in Veneto Region, tasked with procurement, processing, preserving, validating and distributing human tissue for clinical use. During Covid-19 outbreak, operations in FBTV have never stopped and a great effort was required to maintain a standard trend of activity. The aim of this study was to describe the impact of Sars-CoV-2 on the activity of a multitissue bank in Italy. Moreover, we investigated the presence of the virus in tissues retrieved from two Sars-CoV-2 positive cadaver donors. Our survey demonstrated that the transplantation network of Veneto Region has positively reacted to the pandemic scenario, thanks to the effort of all personnel involved. Statistical analyses underlined that most of the activities of the tissue bank were unaffected during the Sars-CoV-2 pandemic.

## Introduction

On December 2019, cases of pneumonia of unknown etiology were detected in Wuhan (China). A new type of coronavirus (novel coronavirus, nCoV), was isolated there on 7 January 2020 and subsequently cases were reported in Thailand and Japan (who.int/emergencies/disease-outbreak-news). Fever and cough were the predominant symptoms, but the onset of severe illness was observed since the beginning of the outbreak and a wide spectrum of severity was observed (Guan et al. 2020). Social distancing measures and isolation of cases and their contact were strategies to control the epidemic (Prem et al. 2020; Lavezzo et al. 2020).

Italy was one of the first Western country in which the virus spread. In fact, on 30th January 2020, the first two cases of Sars-CoV-2 were confirmed. The Italian Government declared the health emergency status on 31th January 2020. Since then, precautionary measures were established in order to contain the coronavirus outbreak and on 9th March 2020 Italy was entirely in a lockdown.

The pandemia affected not only public health but also health-care services worldwide. A cohort study in 22 countries reported an overall decrease of 15.92% of organ transplantation in 2020 compared to 2019 (Aubert et al. 2021). In Italy, organ transplantations in 2020 were 10% less than in 2019, while tissue transplantation was 20% decreased (www.trapianti.salute.gov.it).

Fondazione Banca dei Tessuti di Treviso (FBTV) is a non-profit healthcare organisation tasked with procurement, processing, preserving, validating and distributing human tissue for clinical use.

Set up by the Veneto Regional Authorities, FBTV is specialized in the procurement, processing, storage and distribution of cardiovascular tissue, musculoskeletal tissue, adipose tissue and amniotic membrane. It distributes these homologous tissues throughout Italy and beyond. FBTV provides tissue procurement activities in the hospitals of Veneto Region through direct contact with the Hospital Coordinators and the medical and nursing staff. This unit, composed by a medical Coordinator and one or more nurse, is involved in donor selection, contacts the family and evaluates social and medical anamnesis. These high-qualified personnel are coordinated by Regional Competent Authority—Regional Transplant Center. The network where FBTV performed the retrievals is made up of more than 50 Hospital Transplant Coordinators located in the North and in the Centre of Italy, in fact FBTV is also in charge to retrieve in Friuli Venezia Giulia, Trentino and Marche Region. The synergy created among all these operators has led to a progressive increase in the number of donors and subsequent tissue procurement.

To minimize the risk of disease transmission and guarantee continuation of tissues donation, on 24th February 2021, National Transplant Centre (CNT) had ordered the performance of a specific test for Sars-CoV-2 (nasopharyngeal swab o bronchoalveolar lavage) for each donor of the northern regions of Lombardia, Veneto, Piemonte, Emilia Romagna and Trento. Until then, only anamnestic surveillance of patients travelled in endemic areas was mandatory. According to the pandemia trend, further directives for tissue donor selection were issued in the following months, including the RT-PCR of nasopharyngeal swab sample or bronchiolo-alveolar lavage.

From the beginning of the outbreak to June 2021 only two multitissue donors resulted positive to Sars-CoV-2. Tissue samples were analyzed in order to verify the presence of Sars-COV-2. This number highlight the hard work made by hospital personnel to select the low-risk donors, through a strict anamnesis evaluation and family interview.

Covid pandemic affected donation activities worldwide, especially for living donation due to the lack of elective surgery. Moreover, donations from heart-beating (HB) and non-heart-beating (NHB) donors have recorded a decrease.

The aim of this study was to describe the impact of Sars-CoV-2 on the activity of procurement, processing and distribution of tissues for transplant in the Veneto Region multitissue bank and describe how the transplantation network has reacted to the pandemia. Moreover, we investigated the presence of the virus in tissues retrieved from two Sars-CoV-2 positive cadaver donors.

## Methods

A survey of FBTV activity in 2020 compared to the preceding two years was conducted. Moreover, data of the procurement, distribution and processing of tissue in the first half of 2021 were collected and compared to the first half of 2020 and the first half of the two-years period 2018–2019.

### Statistical analysis

Statistical analyses were performed by SigmaPlot 14 (Systat Software Inc, San Jose, CA) using t-test to compare two groups or Kruskal–Wallis One Way Analysis of Variance on Ranks and using Tukey Test post hoc test for multiple comparisons.

### Detection of Sars-CoV-2 in tissue samples by real time PCR

After the retrieval, tissues were transferred in antibiotic solution made up with Gentamicin, Vancomycin and Meropenem and maintained at + 4 °C following the validated FBTV internal procedures (Serafini et al. 2016; Montagner et al. 2018; Paolin et al. 2018).

Before the analysis, tissues were kept at + 4 °C.

The tissues were manually dissected into few sections of approximately 2 mm and treated over night with MagNA Pure Bacteria Lysis Buffer (Roche). Detection of SARS-CoV-2 RNA was performed by an in-house real-time RT–PCR method, which was developed according the protocol and the primers and probes designed by CDC that targeted the gene encoding nucleocapsid (N2) of SARS-CoV-2 and RNA-dependent RNA polymerase. Real-time RT–PCR assays were performed in a final volume of 25 μl, containing 5 μl of purified nucleic acids, using One Step Real Time kit (Thermo Fisher Scientific) and run on ABI 7900HT Fast Sequence Detection Systems (Thermo Fisher Scientific).

## Results

### Tissues procurement

**Fig. 1:**
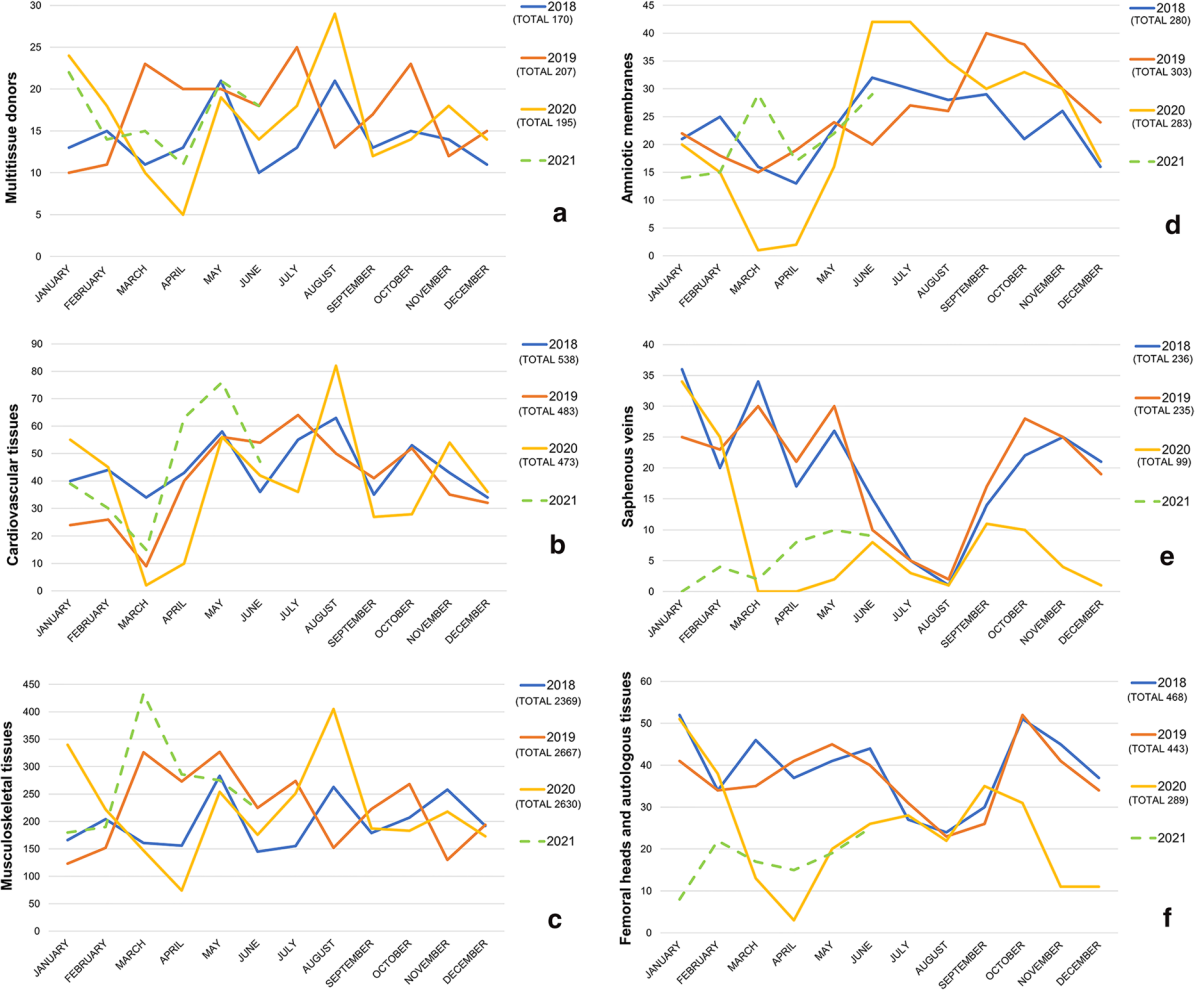
Number of deceased donor (**a**). Cardiovascular (**b**) and musculoskeletal (**c**) tissues procurement from deceased donors. Amniotic membranes (**d**), saphenous veins (**e**), femoral heads and autologous tissues (**f**) procurement from living donations

During 2020 a total of 866 donors were procured (671 living donors and 195 cadaver donors). Tissue procurement from 2018 to the first half of 2021 is depicted in Fig. 1 and Table 1. The procurement of multitissue donors, and consequently of cardiovascular and musculoskeletal tissues, was not significantly affected by the pandemia in 2020. In fact, musculoskeletal ad cardiovascular tissues procurement in 2020, was only slight reduced and the decrease is not significant.

**Table 1:** Procurement

	First 6 months of 2020 versus First 6 months of 2018–2019 (%)	Total 2020 procurement versus 2018–2019	First 6 months of 2021 versus first 6 months of 2018–2019	First 6 months of 2021 versus first 6 months of 2020 (%)
Multitissue donors	− 2.70	+ 3.45%	+ 9.19%	+ 12.22
Musculoskeletal tissues	− 4.53	+ 4.45%	+ 24.83%	+ 30.75
Cardiovascular tissues	− 9.48	− 7.35%	+ 16.38%	+ 28.57
Amniotic membranes	− 22.58	− 2.92%	+ 1.61%	+ 31.25
Saphenous veins	− 51.92	− 57.96%* (*p* = 0.006)	− 77%* (*p* < 0.001)	− 52.17
Femoral heads and autologous tissues	− 38.37	− 36.55%* (*p* = 0.005)	− 56.73%* (*p* < 0.001)	− 29.80

Trend of tissues procurement since the beginning of the outbreak

*Significative difference

In 2020, a significant reduction was observed only in the procurement of tissue from living donors. In fact, saphenous veins procurement decreased by 57.96% compared to the biennium 2018–2019 (*p* = 0.006), while femoral heads and autologous tissue procurement decreased by 36.55% compared to the same period (*p* = 0.005). However, the reduction is significant also in the first semester of 2021 compared to the same period.

### Tissues distribution

**Fig. 2:**
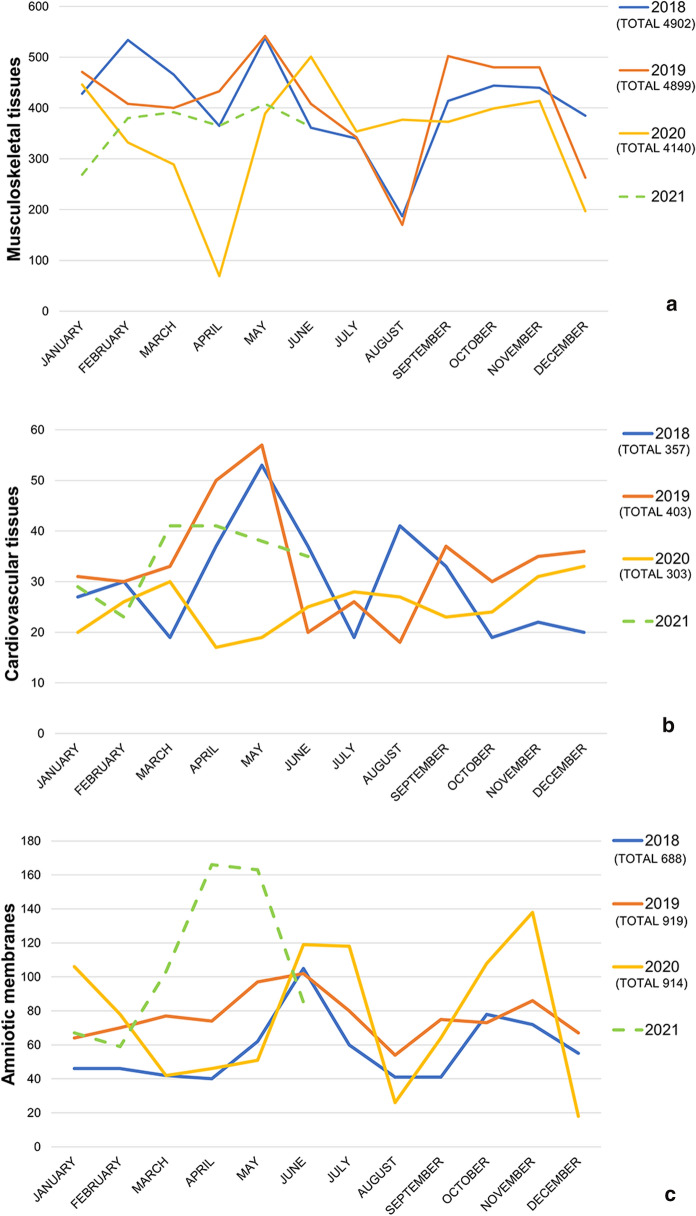
Distribution. Number of musculoskeletal tissues (**a**), cardiovascular tissues (**b**) and amniotic membranes (**c**) distributed from 2018 to the first semester of 2021

Tissues distribution from 2018 to the first half of 2021 is depicted in Fig. 2. The distribution in 2020 was apparently decreased, however, the reduction was significative only in the first semester of 2020 for cardiovascular tissues compared to the biennium 2018–2019 (Table 2). The same trend was found to be significative in the first semester of 2021 compared to the biennium 2018–2019, for musculoskeletal tissues distribution. On the contrary, amniotic membrane distribution significantly increased by 55.88% in the first semester of 2021 compared to the biennium 2018–2019.

**Table 2:** Distribution

	First 6 months of 2020 versus first 6 months of 2018–2019	Total 2020 distribution versus 2018–2019 (%)	First 6 months of 2021 versus first 6 months of 2018–2019	First 6 months of 2021 versus first 6 months of 2020 (%)
Musculoskeletal tissues	− 24.32%	− 15.52	− 18.68% ** (p=0.014)	+ 7.45
Cardiovascular tissues	− 35.38%* (*p* = 0.008)	− 20.26	− 2.36%	+ 51.09
Amniotic membranes	+ 7.15%	+ 13.75	+ 55.88%* (*p* = 0.031)	+ 45.48

Trend of tissues distribution since the beginning of the outbreak

*Significative difference

### Tissues processing

**Fig. 3:**
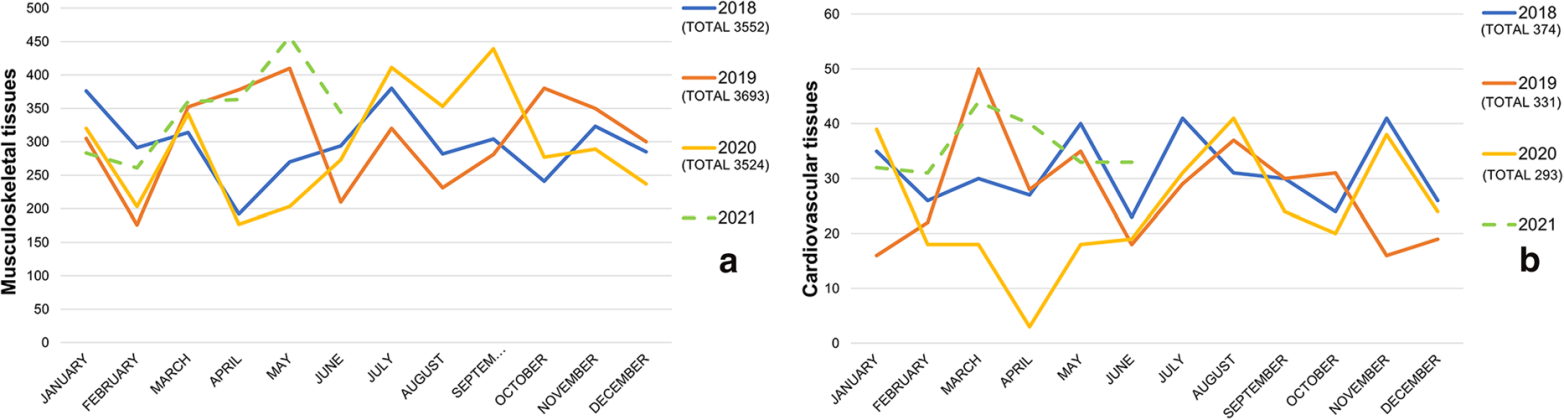
Processing. Number of musculoskeletal (**a**) and cardiovascular (**b**) tissues processed from 2018 to the first semester of 2021

The number of tissues processing in the period 2018-first half of 2021 is depicted in Fig. 3.

As reported in Table 3, no significative difference of activity was revealed by the analysis.

**Table 3:** Processing

	First 6 months of 2020 versus first 6 months of 2018–2019 (%)	Total 2020 processing versus 2018–2019 (%)	First 6 months of 2021 versus first 6 months of 2018–2019 (%)	First 6 months of 2021 versus first 6 months of 2020 (%)
Musculoskeletal tissues	− 14.91	− 2.72	+ 15.92	+ 36.23
Cardiovascular tissues	− 34.29%	− 16.88	+ 21.71	+ 85.22

No significative difference

### Detection of Sars-CoV-2 in tissues samples

Two donors resulted positive to nasopharyngeal swab.

Six tissues of the first positive donor were analyzed: costal cartilage, fascia lata, cardiac tissue, pericardium, gracilis and semitendinosus tendon and anterior tibial tendon. Three tissues of the second positive donor were analyzed: right and left femoral heads and one iliac artery.

PCR analysis of tissue samples retrieved from a Sars-CoV-2 positive donors didn’t detect the viral presence.

## Discussion

In Italy, two waves of Sars-CoV-2 infection have been identified: the first one lasted 157 days (from the 24th of February to the 29th of July 2020), the second one (from the 30th of July 2020 to the 7th of February 2021) lasted 193 days (Ferrante 2021).

During the two waves of infection, elective and non-urgent surgical procedure were stopped in Veneto Region, the first time from 13th March 2020 to 4th May 2020 and the second one from 10th November 2020. During these periods, a reduction in tissues distribution was obviously recorded.

FBTV continuously stayed abreast of directives from National Transplant Centre and updated the management of risk for the procedures impacted by the pandemia. Several actions were established, in order to reduce the risk of Sars-CoV-2 transmission between operators at the work, such as teleworking, job rotation on site and online meetings. Additional safety devices and sanitizations were introduced during the procurement of tissues. In fact, it is the most critical phase, since it is conducted in potential Sars-CoV-2 positive donors. Moreover, the effort to maintain a standard trend of activity was remarkable, especially in the activity of procurement undertaken by the regional transplantation network.

Our survey was conducted with the aim to evaluate the impact of the Sars-CoV-2 outbreak on our activity. In fact, tissue bank activity in the world was strongly affected by the health emergency. The number of cardiovascular tissue donors was significantly lower in 2020 compared to 2019 in the cardiovascular tissue bank of Lombardy. However, the total cardiovascular tissue collected as well as the rate of cardiovascular tissue distribution were not significantly decreased (Mastroiacovo et al. 2021). A drop of tissue donors was recorded in Spain between March and April 2020, without any correlation with the amount of impact of Sars-CoV-2 in the regions. However, tissue requests were fulfilled due to the lower demand for elective operations (Villalba et al. 2020).

Decrease in musculoskeletal tissue distribution in the first half of 2020 was also reported by other tissue establisments in Europe (Garcia-Lopez et al. 2021). Causes of that reduction were the lack of personnel involved in donor selection and of procurement facilities in hospitals. On the contrary, amniotic membrane distribution was not substantially affected (Garcia-Lopez et al. 2021). Moreover, a dramatic reduction of tissue donation activity and tissue distribution in April 2020 compared to 2019 has been reported by other multitissue bank (Piteira et al. 2021).

Our survey demonstrated that the transplantation network of Veneto Region has positively reacted to the pandemic scenario, thanks to the effort of all personnel involved. Statistical analyses underlined that most of the activities of the tissue bank were unaffected during the Sars-CoV-2 pandemic. Tissue procurement and distribution of musculoskeletal, cardiovascular tissues and amniotic membranes showed only slight modifications and musculoskeletal and cardiovascular tissues processing were also unchanged. In detail, the distribution of cardiovascular tissues was reduced in the first semester of 2020 (2018–2019 vs 2020, *p* = 0.008), whereas the distribution of musculoskeletal tissues was reduced in the first semester of the 2021 (2018–2019 vs 2021, *p* = 0.014). On the contrary, amniotic membrane distribution was increased in the first semester of the 2021 (2018–2019 vs 2021, *p* = 0.031).

Otherwise, we observed that tissue procurement from living donors was significantly reduced for saphenous veins (2018–2019 vs 2020, *p* = 0.006 and first semester 2018–2019 vs first semester 2021, *p* < 0.001) and for femoral heads and autologous tissues (2018–2019 vs 2020 *p* = 0.005 and first semester 2018–2019 vs first semester 2021, *p* < 0.001).

Since the beginning of the outbreak, great attention has been focused on social and medical anamnesis evaluation, with the result that, out of 866 total donors procured in 2020, only two multitissue donors resulted positive to nasopharyngeal swab after retrieval. Tissue samples of this donors were analysed, but Sars-CoV-2 was not found.

To date, it is not already stated the probability of Sars-CoV-2 transmission through tissues transplantation. A recent publication reported the absence of Sars-CoV-2 in myocardium and heart valves of two positive donors, but a higher number of samples must be evaluated (Jashari et al. 2021). Nevertheless, there are no evidence of the efficacy of Sars-CoV-2 inactivation with tissue processing phases such as refrigeration, freezing, disinfection or lyophilisation. On the contrary, gamma irradiation seems to be a promising technique to effectively inactivate the virus (Paggiaro et al. 2021). However, since this technique can damage tissues’ structure and properties (Nguyen et al. 2011; Mrázová et al. 2014; Paolin et al. 2016), the gamma irradiation dose able to inactivate Sars-Co-V-2 virus has to be validated.

## Data Availability

The datasets analysed during the current study were collected in Fondazione Banca dei Tessuti di Treviso and will be available on reasonable request.

## References

[CR1] Aubert O, Yoo D, Zielinski D, Cozzi E, Cardillo M (2021). COVID-19 pandemic and worldwide organ transplantation: a population-based study. Lancet Public Health.

[CR2] Ferrante P (2021) The first year of COVID-19 in Italy: incidence, lethality, and health policies. J Public Health Res, 11(1) 10.4081/jphr.2021.2201 PMC8883532 34615342

[CR3] Garcia-Lopez J, Delgadillo J, Vilarrodona A, Querol S, Ovejo J (2021). SARS-CoV-2/COVID-19 pandemic: first wave, impact, response and lessons learnt in a fully integrated Regional Blood and Tissue Bank. A narrative report. Blood Transfus.

[CR4] Guan WJ, Ni ZY, Hu Y, Liang WH, Ou CQ (2020). Clinical characteristics of coronavirus disease 2019 in China. N Engl J Med.

[CR5] Jashari R, Van Esbroeck M, Vanhaebost J, Micalessi I, Kerschen A, Mastrobuoni S (2021). The risk of transmission of the novel coronavirus (SARS-CoV-2) with human heart valve transplantation: evaluation of cardio-vascular tissues from two consecutive heart donors with asymptomatic COVID-19. Cell Tissue Bank.

[CR6] Lavezzo E, Franchin E, Ciavarella C, Cuomo-Dannenburg G, Barzon L (2020). Suppression of a SARS-CoV-2 outbreak in the Italian municipality of Vo'. Nature.

[CR7] Mastroiacovo G, Guarino A, Pirola S, Gennari M, Capriuoli F, Micheli B, Bonomi A, Piccolo G, Dainese L, Polvani G (2021). Cardiovascular tissue banking activity during SARS-CoV-2 pandemic: evolution of national protocols and Lombardy experience. Cell Tissue Bank.

[CR8] Montagner G, Trojan D, Cogliati E, Manea F, Vantini A, Paolin A (2018). Stability analysis of the antibiotic cocktail used by Treviso Tissue Bank Foundation for tissues decontamination. Cell Tissue Bank.

[CR9] Mrázová H, Koller J, Fujeríková G, Babál P (2014). Structural changes of skin and amnion grafts for transplantation purposes following different doses of irradiation. Cell Tissue Bank.

[CR10] Nguyen H, Morgan DAF, Forwood MR (2011). Validation of 11 kGy as a radiation sterilization dose for frozen bone allografts. J Arthroplasty.

[CR11] Paggiaro AO, Carvalho VF, Gemperli R (2021). Effect of different human tissue processing techniques on SARS-CoV-2 inactivation-review. Cell Tissue Bank.

[CR12] Paolin A, Trojan D, Leonardi A, Mellone S, Volpe A, Orlandi A, Cogliati E (2016). Cytokine expression and ultrastructural alterations in fresh-frozen, freeze-dried and γ-irradiated human amniotic membranes. Cell Tissue Bank.

[CR13] Paolin A, Spagnol L, Battistella G, Trojan D (2018). Evaluation of allograft decontamination with two different antibiotic cocktails at the Treviso Tissue Bank Foundation. PLoS ONE.

[CR14] Piteira AR, Bofill-Ródenas AM, Fariñas O, Tabera J, Vilarrodona A (2021). Lessons learned from SARS-CoV-2 pandemic in donation and tissue banking activities: key takeaways. Transplantation.

[CR15] Prem K, Liu Y, Russell TW, Kucharski AJ, Eggo RM, Davies N, Jit M, Klepac P, Centre for the Mathematical Modelling of Infectious Diseases COVID-19 Working Group (2020). The effect of control strategies to reduce social mixing on outcomes of the COVID-19 epidemic in Wuhan, China: a modelling study. Lancet Public Health..

[CR16] Serafini A, Riello E, Trojan D, Cogliati E, Palù G, Manganelli R, Paolin A (2016). Evaluation of new antibiotic cocktails against contaminating bacteria found in allograft tissues. Cell Tissue Bank.

[CR17] Villalba R, Santos S, Martinez MJ, Díaz M, Pevida M, Cemborain A, Casares C, Mirabet V (2020). Analysis of impact on tissue activity during COVID-19 outbreak: a survey of 8 banks in Spain. Cell Tissue Bank.

[CR18] who.int/emergencies/disease-outbreak-news

